# Whole exome sequencing identifies *MRVI1* as a susceptibility gene for moyamoya syndrome in neurofibromatosis type 1

**DOI:** 10.1371/journal.pone.0200446

**Published:** 2018-07-12

**Authors:** Claudia Santoro, Teresa Giugliano, Markus Kraemer, Annalaura Torella, Jan Claudius Schwitalla, Mario Cirillo, Daniela Melis, Peter Berlit, Vincenzo Nigro, Silverio Perrotta, Giulio Piluso

**Affiliations:** 1 Dipartimento della Donna, del Bambino e di Chirurgia Generale e Specialistica, Università degli Studi della Campania “Luigi Vanvitelli”, Naples, Italy; 2 Dipartimento di Medicina di Precisione, Università degli Studi della Campania “Luigi Vanvitelli”, Naples, Italy; 3 Department of Neurology, Alfried Krupp Hospital, Essen, Germany; 4 Department of Neurology, Heinrich-Heine-University, Medical Faculty, Düsseldorf, Germany; 5 Telethon Institute of Genetics and Medicine (TIGEM), Pozzuoli, Italy; 6 Dipartimento di Scienze Mediche, Chirurgiche, Neurologiche, Metaboliche e dell’Invecchiamento, Università degli Studi della Campania “Luigi Vanvitelli”, Naples, Italy; 7 Dipartimento di Pediatria, Università degli Studi di Napoli "Federico II", Naples, Italy; German Cancer Research Center (DKFZ), GERMANY

## Abstract

**Background and purpose:**

Moyamoya angiopathy is a progressive cerebral vasculopathy. The p.R4810K substitution in *RNF213* has previously been linked to moyamoya disease in Asian populations. When associated with other medical conditions, such as neurofibromatosis type 1, this vasculopathy is frequently reported as moyamoya syndrome. Intriguingly, most cases of moyamoya-complicated neurofibromatosis type 1 have been described in Caucasians, inverting the population ratio observed in Asians, although prevalence of neurofibromatosis type 1 is constant worldwide. Our aim was to investigate whether, among Caucasians, additive genetic factors may contribute to the occurrence of moyamoya in neurofibromatosis type 1.

**Methods:**

Whole exome sequencing was carried out on an Italian family with moyamoya-complicated neurofibromatosis type 1 to identify putative genetic modifiers independent of the *NF1* locus and potentially involved in moyamoya pathogenesis. Results were validated in an unrelated family of German ancestry.

**Results:**

We identified the p.P186S substitution (rs35857561) in *MRVI1* that segregated with moyamoya syndrome in both the Italian and German family.

**Conclusions:**

The rs35857561 polymorphism in *MRVI1* may be a genetic susceptibility factor for moyamoya in European patients with neurofibromatosis type 1. *MRVI1* is a functional partner of *ITPR1*, *PRKG1* and *GUCY1A3*, which are involved in response to nitric oxide. Mutations in *GUCY1A3* have been recently linked to a recessive syndromic form of moyamoya with esophageal achalasia.

## Introduction

Moyamoya is a progressive stenosis of the distal intracranial internal carotid artery (ICA), extending to the proximal anterior and middle arteries, with compensatory development of a hazy network of basal collaterals called moyamoya vessels [1, 2]. When the vasculopathy occurs alone, it is named moyamoya disease (MMD). The annual incidence of MMD in the USA, Japan and Europe is 0.086 [3], 0.54 [4] and 0.03 [5] per 100,000, respectively. The existence of hereditary factors that may play a major role in MMD has been suggested by its occasional familial recurrence, and by its higher prevalence in Japanese and Korean populations [6].

The polymorphic p.R4810K substitution in *RNF213* has been associated with MMD, as well as with intracranial artery stenosis/occlusions, in Japanese and Chinese populations with a founder effect [7]. Very recently, and for the first time, the FREX consortium reported rare variants in the C-terminal region of RNF213 associated with moyamoya angiopathy in patients of European ancestry [8]. In addition, a recent genome-wide association study involving a large case/control cohort of Chinese ancestry identified 10 novel susceptibility loci for MMD, further complicating the genetic basis of this condition [9].

When the vasculopathy is associated with other diseases, such as sickle cell disease, Trisomy 21, Alagille syndrome, neurofibromatosis type 1 (NF1) and others, it is named moyamoya syndrome (MMS). Intriguingly, homeostasis and remodeling processes are somehow impaired by the altered function of genes causing the above conditions. Nevertheless, MMS remains a relatively rare feature in all these disorders, suggesting that genetic factors could trigger its development.

A syndromic form of moyamoya associated with esophageal achalasia was reported caused by homozygous mutations in *GUCY1A3*, the soluble receptor of nitric oxide (NO) [10]. MMS was not a fully penetrant feature of the condition, which was, however, characterized by achalasia.

NF1 is an autosomal dominantly inherited neurocutaneous condition with an estimated incidence of 1:3000 that results from germline mutations in *NF1* mapped to 17q11.2, and characterized by a wide inter- and intra-familial phenotypic variability [11].

NF1 patients present an increased risk for a variety of cardiovascular disorders. Life-threatening or fatal vascular events, including cerebral hemorrhage, arterial aneurysms and rupture of large arteries, are described in young adults with NF1 [12].

MMS is the most frequent cerebral arteriopathy in NF1, occurring in about 2–6% of patients and representing a greater risk for those receiving cranial irradiation [13].

Although MMD occurs frequently among Asians, the majority of MMS-NF1 patients are reported in Europe and the USA [14, 15]. However, the prevalence of NF1 is not related to ethnic origin [16]. The existence of an ethnic predisposition with an allele frequency greater in Caucasians than in Asians might explain why most MMS-NF1 patients are Caucasian. This hypothesis is also supported by the rarity of familial cases of MMS-NF1 co-occurrence.

To date, a low penetrance autosomal dominant model of inheritance has been suggested for MMD, with *RNF213* representing a susceptibility factor for MMD mainly among Asians. The occurrence of MMS in patients affected by NF1 might therefore be considered a characteristic of a complex trait disease.

To identify putative genetic modifiers independent of the *NF1* locus and potentially involved in MMS pathogenesis, we carried out a comprehensive genetic study involving a large Italian family with MMS-NF1 co-occurrence in two first cousins.

## Materials and methods

### Patients

**Fig 1:**
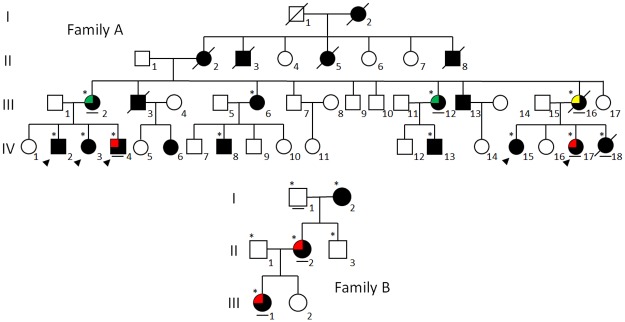
Pedigree of Family A and B with MMS-NF1 co-occurrence. The observed cerebrovascular abnormalities are indicated according to the following pattern fill: moyamoya (red), aneurysm (green) and ectasia (yellow). An asterisk designates subjects recruited for the genetic study: an arrowhead indicates subjects in which WES analysis was carried out, while a bar below the symbol indicates the only family members presenting the *MRVI1* rs35857561 polymorphism.

Family A is a large Italian family being followed at the specialized Pediatric Referral Center for Neurofibromatosis in the Department of Pediatrics of the University of Campania “Luigi Vanvitelli” (Italy). It includes 45 individuals in four generations, 20 of whom are affected by NF1; two first cousins (IV.4 and IV.17) were also diagnosed with MMS in childhood (Fig 1, Tables 1 and 2).

**Table 1:** Clinical and molecular findings associated with neurofibromatosis type 1.

*Family ID*	*Patient ID*	*Sex*	*Age at NF1 diagnosis (yrs)*	*CALMs*	*Freckling*	*Neurofibromas*	*Lisch Nodules*	*Developmental Delay*	*Learning Disability*	*Macrocephaly*	*Other clinical features*	*NF1 inheritance*	*NF1 mutation*
*A*	III.2 (NF394)	F	NA	+	+	Cut(+)	+	-	NA	-	Bullous emphysema	Maternal	c.4515-2A>G (p.R1505Sfs*53)
	III.12 (NF415)	F	NA	+	+	Cut(+)	+	-	NA	-	Noonan-like facies, sternum excavatum	Maternal	
	III.16 (NF477)	F	NA	+	+	Cut(+)	+	-	NA	-	Acute heart failure at 38y, deceased for unknown cause at 41y.	Maternal	
	IV.4 (NF026)	F	0.7	+	+	Cut(+) Ple(-)	+	-	+	-	Severe scoliosis and excavatum sternum, hyperactivity, leg length inequality, hypergonadotropic hypogonadism	Maternal	
	IV.17 (NF262)	M	0.7	+	+	Cut(+) Ple(+)	+	-	+	-	Hyperactivity	Paternal	
B	II.2 (NF440)	F	early childhood	+	+	Cut(+) Ple(-)	+	-	-	-	Accessory breast, hypothyroidism	Maternal	c.480-1G>A (p.?)
	III.1 (NF441)	F	early childhood	+	+	Cut(-), Ple(-)	+	+	+	-	Coxa valga, clubfoot, strabismus, numerous fractures	Maternal	

*Abbreviations*: -, negative; +, positive; Cut, cutaneous/subcutaneous; Ple, plexiform; M, male; F, female; NA, not available.

**Table 2:** Clinical and radiological findings associated with moyamoya and cerebrovascular abnormalities.

*Family ID*	*Patient ID*	*Sex*	*Age at intracranial artery abnormality diagnosis (yrs)*	*Symptoms at onset Indication for MRI execution*	*MMS form*	*Involved vessels*	*Suzuki grading*	*Other cerebrovascular abnormalities*	*Other cardiovascular problems*	*Hemorrhages*, *Infarcts*	*NBOs*	*Therapy*	*Other radiological findings*
A	III.2 (NF394)	F	43	screening				Infundibular aneurysm of left MCA			+		
	III.12 (NF415)	F	42	screening				Ectasia of left ICA (transversal diameter 7.5 mm)			+		
	III.16 (NF477)	F	39	screening				Infundibular aneurysm of right MCA	Acute heart failure	Multiple gliosis	+		
	IV.4 (NF026)	F	10.6	V cranial nerve neurofibroma	Bilateral	uACA, bMCA, uPCA	II-III	-	-	-	+	uMBHS + aspirin	
	IV.17 (NF262)	M	8.5	suspected OPG	Unilateral	uMCA	I	-	-	-	+	uMBHS + aspirin	
B	II.2 (NF440)	F	45	TIA with prickling sensations at the right-sided corner of the mouth lasting for 5 minutes	Bilateral	bMCA	III	-	-	-	-	aspirin	Agenesis of the middle and posterior part of the corpus callosum
	III.1 (NF441)	F	NA	Right hemiparesis	Unilateral	uICA	I	-	-		+		Agenesis of the anterior part of the corpus callosum, gliosis of the left-sided thalamus (NBO?)

*Abbreviations*: ACA, anterior cerebral artery; ICA, internal carotid artery; MBHS, multiple burr-hole surgery; MCA, middle cerebral artery; NBOs, neurofibromatosis brain objects; OPG, optic pathway glioma; PCA, posterior cerebral artery; u, unilateral; b, bilateral.

Family B is being followed at the Alfried-Krupp Hospital (Germany), and includes seven individuals in three generations, three of whom are affected by NF1; one of these cases (III.1) and the mother (II.2) are complicated by MMS (Fig 1, Tables 1 and 2).

A cohort of 41 molecularly characterized NF1 patients was also recruited from clinical centers participating in the study. Magnetic resonance angiography (MRA) data were available for all these unrelated subjects: four showed MMS (NF020, NF066, NF384, NF412) and three presented different cerebrovascular abnormalities (NF106, NF140, NF176), while all the remaining cases exhibited immaculate intracranial arteries (S1 Table). Age at MRA ranged between 2 and 42 years.

For all subjects investigated, diagnosis of MMS was performed according to the diagnostic guidelines for spontaneous occlusion of the circle of Willis [17]. NF1 was clinically and molecularly diagnosed according to NIH criteria [18]. Cerebrovascular findings were collected for all patients included in the study.

The study was approved by the Ethics Committee of the University of Campania “Luigi Vanvitelli” and by the University of Duisburg-Essen (Germany). Written informed consent was obtained from all the subjects recruited.

### DNA extraction

For each subject investigated, blood samples were collected and genomic DNAs were extracted using standard procedures. DNA concentration and purity were assessed using a spectrophotometer (Nanodrop ND 1000, Thermo Scientific) and verified by agarose gel electrophoresis.

### Array CGH analysis

Patient IV.4 was analyzed for chromosomal rearrangements using CGH ISCA v2 8x60K (Agilent Technologies). Labeling and hybridization were performed according to the manufacturer’s specifications. Scanned array images were analyzed by CytoGenomics Software v4.0.3 (Agilent Technologies). To identify copy number variations (CNVs), we used the standard set-up of ADM-2 algorithm, after performing a quality control test. At least four target probes with changes in the number of copies were required for a CNV call. Variants not known to be pathogenic or of doubtful significance were compared with the Database of Genomic Variants (http://dgv.tcag.ca/) and with the DECIPHER database (https://decipher.sanger.ac.uk/) to facilitate their interpretation.

### Whole exome sequencing

Whole exome sequencing (WES) was carried out using HaloPlex Exome Target Enrichment System for Illumina (Agilent Technologies) according to the manufacturer’s instructions. For each sample, 200 ng of genomic DNA was digested with eight different restriction enzymes to create a library of fragments, and hybridized for 16 hours to specific probes for Illumina sequencing. After capture of the biotinylated target DNA using streptavidin beads, nicks in the circularized fragments were closed by a ligase. Finally, the captured target DNA was eluted by NaOH and amplified by PCR. The amplified target molecules were purified using Agencourt AMPure XP beads (Beckman Coulter Genomics).

The enriched target DNA in each library sample was validated and quantified by microfluidic analysis using the Bioanalyzer High Sensitivity DNA Assay Kit and 2100 Bioanalyzer Expert Software (Agilent Technologies). Six single samples were run in a single lane on a HiSeq 1000 System (Illumina), generating 100 bp-long paired end reads.

Generated sequences were analyzed using an in-house pipeline, designed to automate the analysis workflow [19]. Average coverage for all the experiments was 70× and at least 10× for 95% of the target. Paired sequencing reads were aligned to the reference genome (UCSC, hg19 build) using Burrows-Wheeler Aligner, and sorted with SAMtools and Picard (http://picard.sourceforge.net). Calling of single nucleotide variants (SNVs) and small insertions/deletions (Ins/Del) was performed with Genome Analysis Toolkit (GATK) [20] with parameters adapted to the Haloplex-generated sequences. The called SNVs and Ins/Del variants were annotated using ANNOVAR [21], reporting variant position in RefSeq [22], amino acid change, presence in dbSNP v137 [23], frequency in NHLBI Exome Variant Server (http://evs.gs.washington.edu/EVS), 1000 genomes [24] and ExAC browser http://exac.broadinstitute.org) projects, multiple cross-species conservation [25] and prediction scores of damaging on protein activity [26].

### Bioinformatics tools and databases

To highlight potential functional interactions between WES-selected candidate genes and known moyamoya genes/loci, we used STRING (http://string-db.org), a web-based tool for retrieving interacting genes/proteins, providing a critical assessment and integration of protein-protein interactions or functional associations.

Public databases (OMIM, NCBI and PUBMED) were interrogated to obtain a list of human genes related to MMD/MMS that includes causative genes for disorders known to be associated with MMS, as well as genes included in loci already linked to the vasculopathy (S2 Table).

The International Mouse Phenotyping Consortium database (IMPC) (http://www.mousephenotype.org) was interrogated to identify orthologous genes associated with vasculopathic phenotypes resembling moyamoya in potential mouse models predicted by phenotypic similarity (S3 Table).

### Variant validation and mutation screening

The identified rs35857561 polymorphism in *MRVI1* (RefSeq: NM_001098579.2) was genotyped in all the subjects investigated by bidirectional Sanger sequencing. Four sporadic MMS-NF1 patients were also investigated for mutations in *MRVI1*, *GUCY1A3* (RefSeq: NM_000856.5), *PRKG1* (RefSeq: NM_001098512.2) and *ITPR1* (RefSeq: NM_001099952.2) by bidirectional Sanger sequencing of all coding exons (for primer pairs see S4 Table) using BigDye Terminator sequencing chemistry (Life Technologies), and analyzed on an ABI 3130xL automatic DNA sequencer (Life Technologies).

## Results

**Fig 2:**
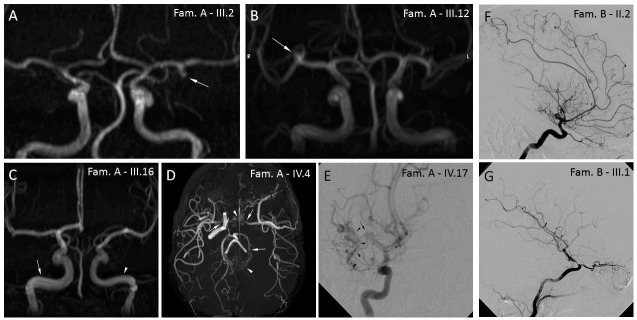
Radiological findings in patients from Family A and B presenting cerebrovascular abnormalities. Family A– 3D MRA without gadolinium MIP reconstructions (A-D) and digital subtraction angiography with catheterization (E): (A) patient III.2 (frontal view), saccular infundibular aneurysm (arrow) of left MCA (M1 tract); (B) patient III.12 (frontal view), saccular infundibular aneurysm (arrow) of right MCA (M1 bifurcation); (C) patient III.16 (frontal view), asymmetry of ICA (intrapetrous and intracavernous tract) with ectasia (arrow) of the right branch (7.5 mm transversal diameter); (D) patient IV.4 (axial view), stenosis/occlusion (arrows) of left ICA, right terminal ICA and left PCA with fine vascular network in the basal cistern, basal ganglia and perimesencephalic cistern compensating for steno-occlusion (moyamoya vessels—arrowheads; Suzuki staging criteria: IV); (E) patient IV.17 (frontal view), stenosis/occlusion (arrow) of right terminal MCA with fine vascular network in the sylvian valley compensating for steno-occlusion (moyamoya vessels—arrowheads; Suzuki staging criteria: III). Family B—Digital subtraction angiography with catheterization (F, G): (F) patient II. 2, occlusion of MCA and moyamoya collaterals; (G) patient III.1, unilateral high-grade stenosis of intracranial tract of ICA without moyamoya collaterals.

We characterized an Italian family (Family A; Fig 1) with 20 subjects affected by NF1. This family included two first cousins (IV.4 and IV.17) with MMS co-occurrence, and is the largest MMS-NF1 family of Caucasian origin described to date. NF1 phenotype was associated with the NM_000267.3:c.4515-2A>G (p.R1505Sfs*53) mutation, detected in all the affected family members recruited for the study (shown with an asterisk in Fig 1). Clinical data, including MMS characteristics in IV.4 and IV.17 and other minor cerebral arteriopathies observed in relatives (III.2, III.12 and III.16), are summarized in Tables 1 and 2, and in Fig 2. As MMS in IV.4 was also associated with hypergonadotropic hypogonadism (Table 1), we first carried out array CGH analysis to investigate the hypothesis of a *BRCC3* loss-of-function mutation [27], identifying a 47,XXY karyotype compatible with patient’s clinical features.

MMS in IV.4 and IV.17, and the other minor cerebral arteriopathies in relatives, might be caused by an additive genetic susceptibility factor with autosomal dominant inheritance and variable phenotype. To investigate this hypothesis, we carried out WES analysis in IV.4 and IV.17, and in three of their relatives (IV.2, IV.3 and IV.15) who also presented NF1 but had a normal MRA. Data filtering criteria for variants that passed quality controls are summarized in S1 Fig.

IV.4 and IV.17 did not share any of the common or rare variants in *RNF213* already associated with moyamoya angiopathy [7, 8]. We therefore only considered heterozygous variants (mean coverage ≥ 10 reads) with a frequency in the global population ≤ 5% and present in IV.4 and IV.17, but absent in relatives analyzed. Synonymous variants were also excluded.

After filtering, 12 variants in 10 genes with no evidence of any causal role in MMS or other cerebral arteriopathies were selected (Table 3).

**Table 3:** List of selected variants after filtering of WES data.

Gene	Full Gene Name	DNA change	Hg19 Chromosome location	Protein change	Exonic Func	dbSNP137	MAF from ExAC Browser
*GIGYF2*	GRB10 Interacting GYF Protein 2	3594_3595insCAG	chr2:233712209	R1198delinsRQ	nonframeshift insertion		
		T3608C	chr2:233712223	L1203P	nonsynonymous SNV		
		A3620C	chr2:233712235	Q1207P	nonsynonymous SNV	rs200557434	NA
*CCDC179*	Coiled-Coil Domain Containing 179	G37A	chr11:22881931	V13I	nonsynonymous SNV	rs145779712	0.001514
*PPFIBP2*	PTPRF Interacting Protein, Binding Protein 2 (Liprin Beta 2)	G1258A	chr11:7669658	V420M	nonsynonymous SNV		
*ANKRD33*	Ankyrin Repeat Domain 33	A796C	chr12:52285101	I266L	nonsynonymous SNV	rs202069826	0.0004152
*DRD5*	Dopamine Receptor D5	C262T	chr4:9783915	L88F	nonsynonymous SNV	rs148402761	0.004521
*SLIT3*	Slit Homolog 3 (Drosophila)	C3341G	chr5:168112927	P1114R	nonsynonymous SNV	rs35305517	0.01529
*AQP6*	Aquaporin 6, Kidney Specific	G700A	chr12:50369305	V234I	nonsynonymous SNV	rs17124220	0.06377
***MRVI1***	**Murine Retrovirus Integration Site 1 Homolog**	**C556T**	**chr11:10650367**	**P186S**	**nonsynonymous SNV**	**rs35857561**	**0.07232**
*SSPO*	SCO-Spondin	C5644T	chr7:149489491	R1882C	nonsynonymous SNV	rs1076277	0.08877
*CHD6*	Chromodomain Helicase DNA Binding Protein 6	C3614G	chr20:40079655	A1205G	nonsynonymous SNV	rs41278126	0.03144

We then generated two lists of genes potentially associated with MMD/MMS: the first included all human genes at *MMD/MMS* loci selected on the basis of current knowledge (S2 Table); the second included all genes related to a vasculopathic phenotype resembling moyamoya in potential mouse models predicted by searching for phenotypic similarity in the IMPC database (S3 Table). A total of 200 genes were selected: 130 human and 70 mouse.

**Fig 3:**
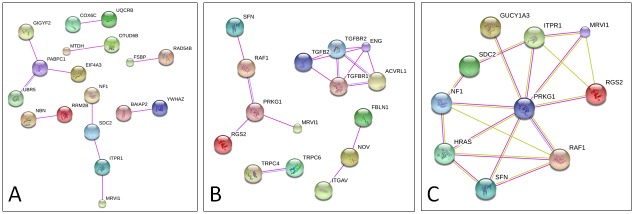
Identification of MRVI1, GUCY1A3, PRKG1and ITPR1 pathways by STRING analysis. Functional interaction networks obtained from human gene list (A) showing *MRVI1* interaction with *ITPR1*, and from murine gene list (B) highlighting *MRVI1* interaction with *Prkg1*. (C) *MRVI1*, *ITPR1* and *PRKG1* are indirectly associated with *GUCY1A3* and *NF1*, as well as with other members of Ras/MAPK signaling pathway. Colored lines indicate known interactions from curated databases (blue), experimentally determined interactions (magenta) and text mining (green). All genes not showing interactions were omitted.

Both lists were separately merged with the WES-selected genes. A search for experimentally validated functional interactions using STRING database detected the interaction networks shown in Fig 3. Among WES-selected genes, only *MRVI1* was found to interact with *ITPR1* (Fig 3A), located in the human *MYMY1* locus, and with *Prkg1*, present in the list of moyamoya-like phenotypes in mouse models (Fig 3B). *PRKG1* is mutated in familial autosomal dominant inherited aortic aneurysm [28]. Interestingly, *MRVI1*, *ITPR1* and *PRKG1* are also indirectly linked to *GUCY1A3* and *NF1*, as well as to other members of Ras/MAPK signaling pathway (Fig 3C).

The nonsynonymous variant found in *MRVI1* is the NM_001098579.2:c.556C>T (p.P186S) substitution, and corresponds to the single nucleotide polymorphism rs35857561 (Table 3). The minor allele frequency reported in ExAC browser for rs35857561 was 0.0706 in Caucasians versus 0.0026 in Asians.

### MRVI1 variant validation

The identified *MRVI1* functional pathway is involved in response to NO [29], as is *GUCY1A3*, which encodes the major NO receptor, mutations of which have been recently associated with MMS and esophageal achalasia [10]. Therefore, the rs35857561 polymorphism in *MRVI1* was first validated in index cases and subsequently by studying its segregation in the 12 subjects from Family A it had been possible to recruit.

As expected, rs35857561 segregated with MMS and other vasculopathies in five out of the 12 family members investigated (Fig 1, bar below the symbol), while it was absent in six of those with normal MRA. Only patient IV.18 showed the rs35857561 variant despite a normal MRA. However, MRA was performed at 3 years of age, too early to definitively exclude the risk of developing a vasculopathy in later life.

We then extended the study to an additional MMS-NF1 family of German ancestry (Family B; Fig 1).

For Family B, NF1 phenotype was associated with the NM_000267.3:c.480-1G>A mutation, detected in all the affected family members recruited for the study. The index case (III.1) and her mother (II.2) both showed MMS-NF1 co-occurrence (Fig 2 and Tables 1 and 2). Interestingly, the rs35857561 polymorphism in *MRVI1* was likewise identified in both moyamoya-complicated NF1 cases (II.2 and III.1) but was absent in the 80-year-old maternal grandmother (I.2), who suffered NF1 without any type of cerebral vasculopathy.

We subsequently genotyped a further cohort of 41 molecularly diagnosed NF1 patients with available MRA data (S1 Table); four (NF020, NF066, NF384, NF421) presented MMS, and three others (NF106, NF140, NF176) were affected by different abnormalities of intracranial arteries. None of the seven NF1 patients with a positive MRA presented the rs35857561 polymorphism, while only four of the subjects with a negative MRA presented the variant. However, these patients were younger than 18 years when they underwent neuroimaging.

### *MRVI1*, *GUCY1A3*, *PRKG1 and ITPR1* sequencing in sporadic MMS-NF1 patients

*MRVI1* and its functional partners *ITPR1*, *PRKG1* and *GUCY1A3* play an important role in response to NO [30]. In addition, mutations in *PRKG1* can cause thoracic aortic aneurysms and acute aortic dissections [28], while mutations in *GUCY1A3* are associated with an autosomal-recessive disease leading to severe moyamoya and early-onset achalasia [10].

Sporadic *MMS-NF1* patients (NF020, NF066, NF384 and NF421) were therefore screened for variants in *MRVI1* different from the rs35857561 polymorphism, as well as for variants in the functionally related genes *ITPR1*, *PRKG1* and *GUCY1A3*. All coding exons of these genes were sequenced, but no recurrent variants or mutations with a potential functional effect were identified.

## Discussion

Here we report the results of a comprehensive genetic study in an Italian family with MMS-NF1 co-occurrence, the largest Caucasian family described to date, in which WES analysis identified the p.P186S substitution in *MRVI1* co-segregating with MMS and other cerebral arteriopathies. The genetic association was replicated in an unrelated MMS-NF1 family of German ancestry, but not in a very small cohort of 41 NF1 patients with MRA data.

Very recently, rare missense variants in the C-terminal region of RNF213 were associated with moyamoya angiopathy in patients of European ancestry [8]. However, a low proportion (less than 25%) of mutant alleles was detected in MMD and MMS groups, strongly suggesting the contribution of additional genetic factors [8]. None of the *RNF213* variants reported by Guey S. *et al*. or other rare variants were found in our family.

As reported for MMD in Asians, genetic predisposing factors other than *RNF213* exist [9, 31–34], and *MRVI1* may be involved in MMS-NF1 co-occurrence in Europeans.

*MRVI1* is a functional partner of other genes (*ITPR1*, *PRKG1* and *GUCY1A3*), differently related to MMS and other vasculopathies, but together involved in response to NO [30]. NO signaling regulates vascular tone and causes endothelium-dependent vasodilation. *GUCY1A3*, the disease-causing gene in a recessive form of MMS with esophageal achalasia [10], is the main soluble receptor for NO.

The 11p15.4 cytoband, where *MRVI1* is located, has not been previously linked to familial MMD among Asians. Conversely, this region was recently linked to retinal vessel diameter, with the *D11S1999* marker closely located to the 5’UTR of *MRVI1* [35].

*MRVI1* comprises 21 known exons, and is one of the most complex human genes in terms of transcriptional and post-transcriptional regulation, with a total of 36 *MRVI1* isoforms [29]. The p.P186S substitution is located in exon 5 of *MRVI1*, which is expressed in all known variants.

*MRVI1* encodes for IP3R-associated cGMP kinase substrate, and is assembled in a macrocomplex with cGKI (encoded by *PRKG1*) and IP3RI [36].

cGKI exists as two distinct variants, cGKIα and cGKIβ; cGKIβ selectively binds *MRVI1*, phosphorylating the serine at position 696 [36]. In response to NO, *MRVI1* induces the relaxation of smooth muscle cells of the colon and aorta.

Mutations in *GUCY1A3*, encoding the α1 subunit of soluble guanylyl cyclase, cause an autosomal recessive form of esophageal achalasia, in which MMS is present with incomplete penetrance [10]. *MRVI1* knockout mice display a phenotype that partially overlaps that observed in humans with homozygous mutations of *GUCY1A3*, with the presence of dilated intestine and stomach in sacrificed animals [37]. Intracranial vessels were not investigated in the study.

*MRVI1* also plays an important role in platelet adhesion, secretion and aggregation. The rs7940646 polymorphism in *MRVI1* was previously associated with higher platelet aggregation in response to agonists in humans [38], confirming its role in platelet function.

These observations, together with the genetic findings presented here, make *MRVI1* an attractive genetic susceptibility factor for MMS in NF1 patients. We hypothesize that the p.P186S polymorphism in *MRVI1* might play an additional pathogenetic role respect of *NF1* in such cohort of patients leading to MMS.

The p.P186S substitution segregates with MMS and minor intracranial vascular dilatations in both our families. Only one discordant genotype was found in Family A, in which the child (IV.16) carried the variant but had a normal MRA. However, MRA had been performed at 3.4 years of age, when vascular abnormalities might not yet be detectable, possibly explaining the observed discordance. This patient recently died from sepsis during third-line chemotherapy for optic pathway glioma. Incomplete penetrance is perhaps not unexpected in moyamoya angiopathy. Guey S. *et al*. reported that only 25% of relatives carrying rare missense RNF213 mutations resulted clinically affected, suggesting that additional genetic factors were most likely shared in families [8]. Similarly, incomplete penetrance of moyamoya was reported for *GUCY1A3* mutation carriers [10]. Very recently, reduced penetrance was also observed for *CCER2*, a novel candidate gene for MMD identified by WES analysis [39].

### Pathogenetic hypothesis

The p.(P186S) substitution identified in *MRVI1* is located in exon 5, which is conserved in all the known isoforms of *MRVI1*. This amino acid change might perturb the phosphorylation status of the protein, which is constitutionally phosphorylated at Ser367 and activated through phosphorylation of Ser657 in presence of cGMP and NO [30]. Experimental use of the PRKG1 inhibitor also prevents phosphorylation of Ser657 and Ser670 in response to NO and cGMP. All serine residues susceptible to phosphorylation are shown in S2A Fig. Scansite analysis (http://scansite.mit.edu/) of *MRVI1* protein product (isoform 1) showed that the identified p.(P186S) substitution could disrupt accessibility of the S189 phosphorylation site (S2B Fig).

Neurofibromin, the protein product of *NF1*, regulates the Ras-induced reactive oxygen species production, predisposing NF1 patients to occlusive arterial disease [40, 41]. Since heterozygous *NF1* mutations make vessels prone to stenosis after a damage, co-occurrence of the p.(P186S) substitution identified in *MRVI1* might play a trigger role in this scenario, perturbing relaxation of arteries.

## Conclusions

To our knowledge this is the first study that investigates Caucasian NF1 families with MMS co-occurrence by WES. We provide genetic evidence that, via its rs35857561 polymorphism, *MRVI1* might represent a genetic susceptibility factor for moyamoya angiopathy in NF1 patients of European ancestry. However, the contribution of other genes cannot be excluded, considering the high number of loci already associated with moyamoya in Asians.

Results presented here suggest focusing on cell response to NO. As *GUCY1A3* and *PRKG1* have already been clearly linked to MMS with esophageal achalasia and inherited aortic aneurysm respectively, the role of NO pathway in moyamoya pathogenesis warrants further investigation.

## Supporting information

S1 Fig Variant data filtering. Criteria for step-by-step filtering and resulting number of variants.
(DOCX)

S2 Fig Detection of putative phosphorylation sites of MRVI1 using ScanSite. (A) Phosphorylation sites detected in *MRVI1* protein product (isoform 1; Q9Y6F6). (B) In presence of the p.(P186S) substitution, S189 phosphorylation site is lost compared to wild type.
(DOCX)

S1 Table Radiological findings and rs35857561 genotype for the extended cohort of MRA-screened NF1 patients. (DOCX)

S2 Table List of human genes already associated with MMS or MMD (in loci linked to familial MMD, causative of diseases at risk of MMS, causative of specific syndromes with co-occurrence of MMS). (DOCX)

S3 Table List of mouse genes predicted to be potentially causative of moyamoya disease by phenotypic similarity at IMPC with PhenoDigm score in decreasing order. (DOCX)

S4 Table List of PCR primers with Hg19 genomic coordinates of amplicons for MRVI1 (RefSeq: NM_001098579.2), GUCY1A3 (RefSeq: NM_000856.5), PRKG1 (RefSeq: NM_001098512.2) and ITPR1 (RefSeq: NM_001099952.2). (DOCX)
