# Supplementary material for: Whole exome sequencing identifies MRVI1 as a susceptibility gene for moyamoya syndrome in neurofibromatosis type 1
Source: PLoS One. 2018 Jul 12;13(7):e0200446. doi: 10.1371/journal.pone.0200446 (PMC6042724; doi:10.1371/journal.pone.0200446)

**S1 Fig. Variant data filtering.** Criteria for step-by-step filtering and resulting number of variants


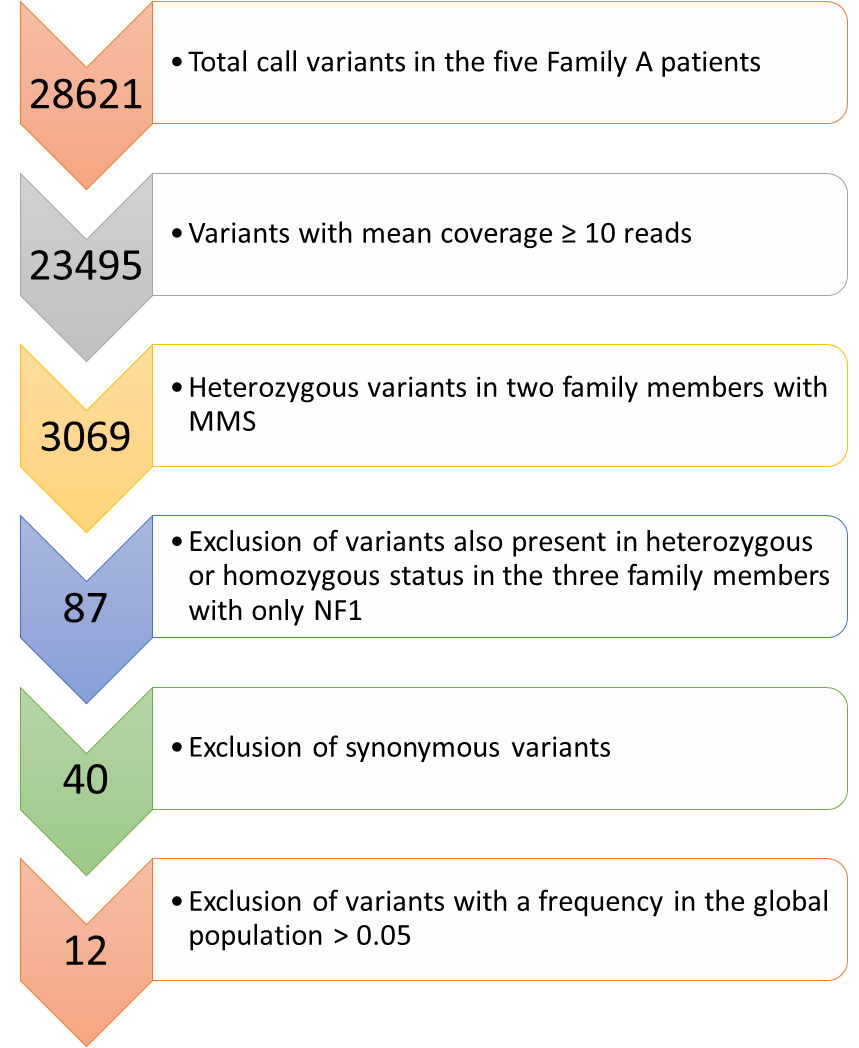

Supplement: S1 Fig — Criteria for step-by-step filtering and resulting number of variants. (DOCX) [file pone.0200446.s001.docx]
