# Supplementary material for: Whole exome sequencing identifies MRVI1 as a susceptibility gene for moyamoya syndrome in neurofibromatosis type 1
Source: PLoS One. 2018 Jul 12;13(7):e0200446. doi: 10.1371/journal.pone.0200446 (PMC6042724; doi:10.1371/journal.pone.0200446)
Supplement: S2 Fig — (A) Phosphorylation sites detected in MRVI1 protein product (isoform 1; Q9Y6F6). (B) In presence of the p.(P186S) substitution, S189 phosphorylation site is lost compared to wild type. (DOCX) [file pone.0200446.s002.docx]

**S2 Fig. Detection of putative phosphorylation sites of MRVI1 using ScanSite.** (A) Phosphorylation sites detected in *MRVI1* protein product (isoform 1; Q9Y6F6). (B) In presence of the p.Pro186Ser substitution, S189 phosphorylation site is lost compared to wild type.

**
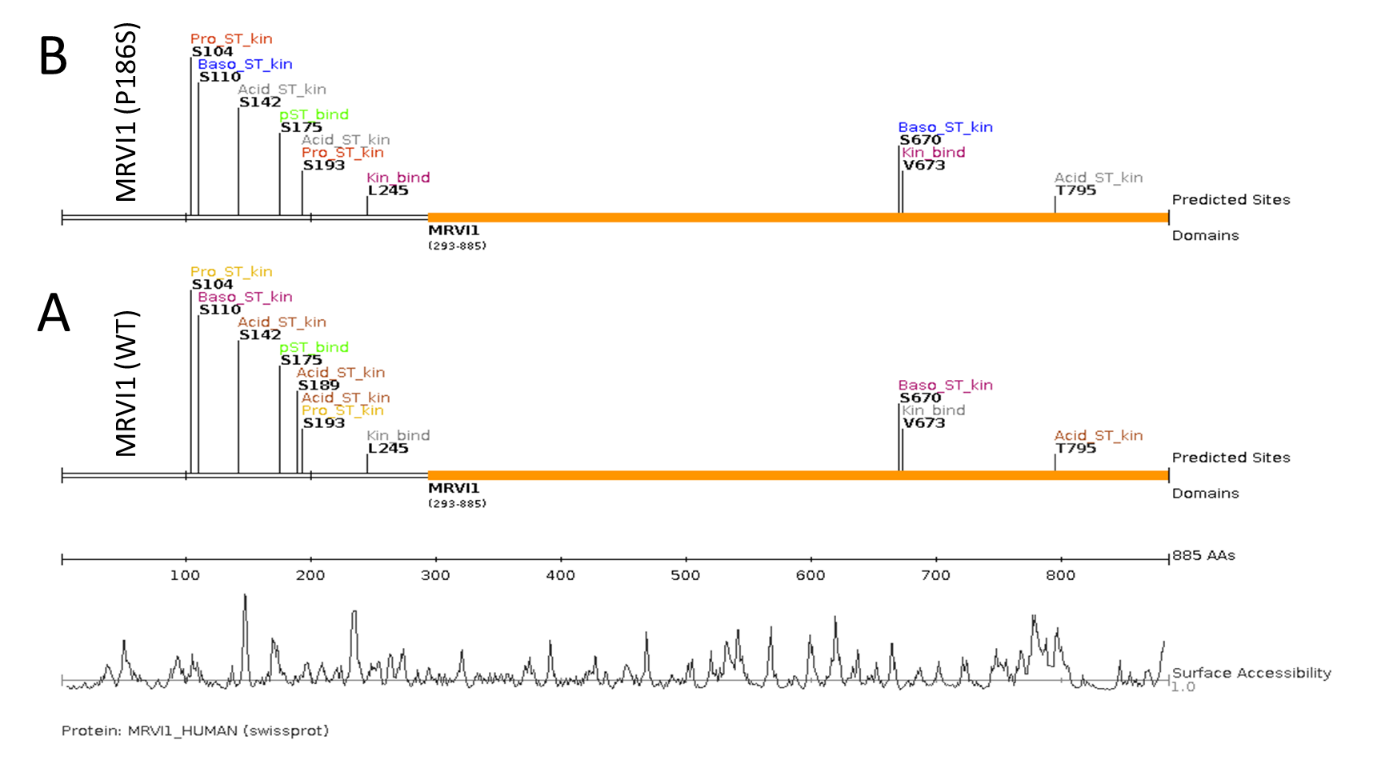
**
