# Supplementary material for: Whole exome sequencing identifies MRVI1 as a susceptibility gene for moyamoya syndrome in neurofibromatosis type 1
Source: PLoS One. 2018 Jul 12;13(7):e0200446. doi: 10.1371/journal.pone.0200446 (PMC6042724; doi:10.1371/journal.pone.0200446)
Supplement: S1 Table — (DOCX) [file pone.0200446.s003.docx]

**S1 Table. Radiological findings and rs35857561 genotype for the extended cohort of MRA-screened NF1 patients.**

| **Patient ID** | **MRA pathological findings** | **Age at MRA (yrs)** | **rs35857561 in *MRVI1*** |
| --- | --- | --- | --- |
| NF006 | - | 3.6 | - |
| NF008 | - | 9 | - |
| NF014 | - | 22 | - |
| NF020 | MMS with unilateral occlusion of MCA | 10.3 | - |
| NF037 | - | 9.2 | - |
| NF049 | - | 19 | + |
| NF062 | - | 4 | - |
| NF065 | - | 3.5 | - |
| NF066 | MMS with bilateral occlusion of MCA and ACA | 14 | - |
| NF070 | - | 5.10 | - |
| NF076 | - | 3.1 | - |
| NF085 | - | 7.2 | - |
| NF088 | - | 5.3 | - |
| NF097 | - | 20 | - |
| NF101 | - | 7.11 | - |
| NF106 | Occlusion of right ICA at its origin with secondary ectasia of right ICA, plus aneurism of the right A1-A2 junction | 3 | - |
| NF108 | - | 9 | - |
| NF125 | - | 1.2 | - |
| NF128 | - | 2.10 | + |
| NF130 | - | 16 | - |
| NF132 | - | 1 | - |
| NF140 | Infundibulum of the left anterior choroidal artery | 2 | - |
| NF145 | - | 9.1 | - |
| NF160 | - | 2.11 | - |
| NF161 | - | 11.3 | + |
| NF162 | - | 6.4 | - |
| NF173 | Ectasia of the left ICA | 6.2 | - |
| NF216 | - | 6.8 | - |
| NF220 | - | 17 | - |
| NF253 | - | 6 | - |
| NF276 | - | 1.8 | - |
| NF286 | - | 7.3 | - |
| NF291 | - | 5.6 | - |
| NF300 | - | 38 | - |
| NF325 | - | 10.11 | - |
| NF358 | - | 1.6 | - |
| NF384 | MMS with bilateral occlusion of MCA and ACA | NA | - |
| NF421 | MMS with unilateral occlusion of ICA and MCA | 42 | - |
| NF470 | - | 3 | - |
| NF481 | - | 8.3 | - |
| NF500 | - | 2 | + |

*Abbreviations:* MMS, Moyamoya syndrome; ACA, anterior cerebral artery; MCA, middle cerebral artery; ICA, internal carotid artery; NA, not available.
