# Supplementary material for: Whole exome sequencing identifies MRVI1 as a susceptibility gene for moyamoya syndrome in neurofibromatosis type 1
Source: PLoS One. 2018 Jul 12;13(7):e0200446. doi: 10.1371/journal.pone.0200446 (PMC6042724; doi:10.1371/journal.pone.0200446)
Supplement: S2 Table — (DOCX) [file pone.0200446.s004.docx]

**S2 Table. List of human genes already associated with MMS or MMD (in loci linked to familial MMD, causative of diseases at risk of MMS, causative of specific syndromes with co-occurrence of MMS).**

| **Locus/Disease name** | **Chr** | **Genes** | **Reference** |
| --- | --- | --- | --- |
| MYMY1 | 3p26-p24.2 | CNTN6, CNTN4, CNTN4-AS2, CNTN4-AS1, IL5RA, TRNT1, CRBN, LRRN1, SETMAR, SUMF1, ITPR1-AS, ITPR1, EGOT, BHLHE40-AS1, BHLHE40, EDEM1, MIR4890, LOC101927347, GRM7 | [[1](#_ENREF_1)] |
| MYMY2 | 17q25.3 | RNF213 | [[2](#_ENREF_2)] |
| MYMY3 | 8q21.3-q22.3 | SLC7A13, WWP1, RMDN1, CPNE3, CNGB3, CNBD1, DCAF4L2, MMP16, RIPK2, OSGIN2, NBN, DECR1,CALB1, LINC00534, LINC01030, TMEM64, NECBA1, Corf88, TMEM55A, OTUDSB-AS1, OTUD6B, LRRC69, MIR4661, SLC26A7, RUNX1T1, FLJ46284, TRIQK, MIR8084, C8orf87, LINC00535, FAM92A1, RBM12B, RBM12B-AS1, TMEM67, MIR378D2, PDP1, CDH17, GEM, RAD54B, FSBP, KIAA1429, LOC100288748, ESRP1, DPY19L4, INTS8, CCNE2, TP53INP1, NDUFAF6, MIR3150B, MIR3150A, PLEKHF2, C8orf69, C8orf37, C8orf837-AS1, LOC100500773, GDF6, UQCRB, MTERF3, PTDSS1, SDCD2, CPQ, TSPYL5, MTDH, LAPTM4B, MATN2, RPL30, SNORA72, ERICH5, HRSP12, POP1, NIPAL2, KCNS2, STK3, OSR2, VPS13B, MIR599, MIR875, COX6C, RGS22, FBXO43, POLR2K, SPAG1, RNF19A, MIR4471, ANKRD46, SNX31, PABPC1, MIR7705, YWHAZ, FLJ42969, ZNF706, NAKAP1, GRHL2, NCALD, MIR5680, RRM2B, UBR5, ODF1, KLF10, AZIN1, ATP6V1C1, BAALCOS, BAALC, MIR31B1, BAAL-AS1, FZD6, CTHRC1, SLC25A32, DCAF13, RIMS2, DCSTAMP, DPYS, LRP12 | [[3](#_ENREF_3)] |
| MYMY4 | Xq28 | BRCC3, MTCP1NB | [[4](#_ENREF_4)] |
| MYMY5 | 10q23.31 | ACTA2 | [[5](#_ENREF_5)] |
| MYMY6 | 4q32.1 | GUCY1A3 | [[6](#_ENREF_6)] |
| MICROCEPHALIC OSTEODYSPLASTIC PRIMORDIAL DWARFISM, TYPE II; MOPD2 | 21q22.3 | PCNT | [[7](#_ENREF_7)] |
| IMMUNOOSSEOUS DYSPLASIA, SCHIMKE TYPE | 2q35 | SMARCAL1 | [[8](#_ENREF_8)] |
| ALAGILLE SYNDROME 1; ALGS1 | 20p12.2 | JAG1 | [[9](#_ENREF_9)] |
|  | 1p12 | NOTCH2 | [[10](#_ENREF_10)] |
| NEUROFIBROMATOSIS, TYPE I; NF1 | 17q21 | NF1 |  |
| AORTIC ANEURYSM, FAMILIAL THORACIC 6; AAT6 | 10q23.31 | ACTA2 | [[5](#_ENREF_5)] |
| SC PHOCOMELIA SYNDROME | 8p21.1 | ESCO2 | [[11](#_ENREF_11)] |
| HEMOGLOBIN--BETA LOCUS; HBB METHEMOGLOBINEMIA, BETA-GLOBIN TYPE | 11p15.4 | HBB |  |
| Personal case report | 20q11.3 | RALGAPB, ADIG, ARHGAP40, SLC32A1, ACTR5 | [[12](#_ENREF_12),[13](#_ENREF_13)] |

**Supplementary References**

1. Ikeda H, Sasaki T, Yoshimoto T, Fukui M, Arinami T (1999) Mapping of a familial moyamoya disease gene to chromosome 3p24.2-p26. Am J Hum Genet 64: 533-537.

2. Mineharu Y, Liu W, Inoue K, Matsuura N, Inoue S, et al. (2008) Autosomal dominant moyamoya disease maps to chromosome 17q25.3. Neurology 70: 2357-2363.

3. Sakurai K, Horiuchi Y, Ikeda H, Ikezaki K, Yoshimoto T, et al. (2004) A novel susceptibility locus for moyamoya disease on chromosome 8q23. J Hum Genet 49: 278-281.

4. Miskinyte S, Butler MG, Herve D, Sarret C, Nicolino M, et al. (2011) Loss of BRCC3 deubiquitinating enzyme leads to abnormal angiogenesis and is associated with syndromic moyamoya. Am J Hum Genet 88: 718-728.

5. Guo DC, Papke CL, Tran-Fadulu V, Regalado ES, Avidan N, et al. (2009) Mutations in smooth muscle alpha-actin (ACTA2) cause coronary artery disease, stroke, and Moyamoya disease, along with thoracic aortic disease. Am J Hum Genet 84: 617-627.

6. Hervé D, Philippi A, Belbouab R, Zerah M, Chabrier S, et al. (2014) Loss of alpha1beta1 soluble guanylate cyclase, the major nitric oxide receptor, leads to moyamoya and achalasia. Am J Hum Genet 94: 385-394.

7. Rauch A, Thiel CT, Schindler D, Wick U, Crow YJ, et al. (2008) Mutations in the pericentrin (PCNT) gene cause primordial dwarfism. Science 319: 816-819.

8. Boerkoel CF, Takashima H, John J, Yan J, Stankiewicz P, et al. (2002) Mutant chromatin remodeling protein SMARCAL1 causes Schimke immuno-osseous dysplasia. Nat Genet 30: 215-220.

9. Oda T, Elkahloun AG, Pike BL, Okajima K, Krantz ID, et al. (1997) Mutations in the human Jagged1 gene are responsible for Alagille syndrome. Nat Genet 16: 235-242.

10. McDaniell R, Warthen DM, Sanchez-Lara PA, Pai A, Krantz ID, et al. (2006) NOTCH2 mutations cause Alagille syndrome, a heterogeneous disorder of the notch signaling pathway. Am J Hum Genet 79: 169-173.

11. Schule B, Oviedo A, Johnston K, Pai S, Francke U (2005) Inactivating mutations in ESCO2 cause SC phocomelia and Roberts syndrome: no phenotype-genotype correlation. Am J Hum Genet 77: 1117-1128.

12. Santoro C, Malan V, Bertoli M, Boddaert N, Vidaud D, et al. (2013) Sporadic NF1 mutation associated with a de-novo 20q11.3 deletion explains the association of unusual facies, Moyamoya vasculopathy, and developmental delay, reported by Bertoli et al. in 2009. Clin Dysmorphol 22: 42-43.

13. Bertoli M, Boddaert N, Raoul O, Amiel J, Lyonnet S (2009) Sporadic case of unusual facies, cerebral vascular anomalies and developmental delay. Clin Dysmorphol 18: 110-111.
