# Supplementary material for: Whole exome sequencing identifies MRVI1 as a susceptibility gene for moyamoya syndrome in neurofibromatosis type 1
Source: PLoS One. 2018 Jul 12;13(7):e0200446. doi: 10.1371/journal.pone.0200446 (PMC6042724; doi:10.1371/journal.pone.0200446)
Supplement: S3 Table — (DOCX) [file pone.0200446.s005.docx]

**S3 Table. List of mouse genes predicted to be potentially causative of Moyamoya disease by phenotypic similarity at IMPC with PhenoDigm score in decreasing order.**

| **Mouse phenotype**  **Orthologous genes** | **MGI Mouse Phenotype Evidence (PhenoDigm) (x10)** | **Mouse phenotype**  **Orthologous genes** | **MGI Mouse Phenotype Evidence (PhenoDigm) (x10)** |
| --- | --- | --- | --- |
| **MYMY1** | | | |
| Il1rn | 8.486 | Mtap | 7.1 |
| Eng | 7.957 | Uts2r | 7.073 |
| Pecam1 | 7.903 | Smtnl1 | 7 |
| Adm | 7.866 | Cbs | 6.894 |
| Ptgdr | 7.739 | Cbs | 6.894 |
| Rgs2 | 7.636 | Chrm5 | 6.893 |
| Appl1 | 7.636 | P2ry6 | 6.885 |
| Ptprm | 7.628 | Ptger3 | 6.838 |
| Trpc4 | 7.628 | Acvrl1 | 6.785 |
| Nos3 | 7.628 | Asic2 | 6.772 |
| Zeb1 | 7.415 | Gucy1a3 | 6.712 |
| Kcna5 | 7.40 | Prkg1 | 6.7 |
| Nox1 | 7.379 | Slc4a7 | 6.659 |
| Klf15 | 7.254 | Il10 | 6.64 |
| Trpc6 | 7.193 | Ptger2 | 6.576 |
| Ramp1 | 7.193 | Agxt2 | 6.569 |
| Nov | 7.181 | Tlr3 | 6.531 |
| Sorl1 | 7.181 | Hmox1 | 6.531 |
| Csrp2 | 7.181 | Mmp9 | 6.531 |
| Kcnmb1 | 7.114 | Kcnk6 | 6.528 |
| **MYMY5** | | | |
| Acta2 | 4.916 | Ednra | 7.19 |
| Tgfb2 | 8.802 | Ece1 | 7.184 |
| Atp7a | 7.708 | Mus81 | 6.853 |
| Tgfbr2 | 7.395 | Hectd1 | 6.695 |
| Tgfbr1 | 7.216 | Mkl2 | 6.265 |
| **MYMY4** | | | |
| Alkbh1 | 7.477 | Itgav | 6.793 |
| Tgfb2 | 7.183 | Plat | 6.768 |
| Fbln1 | 7.147 | Vkorc1 | 6.762 |
| Ptpn11 | 7.043 | Sfn | 6.716 |
| Nme5 | 6.923 | Ehd1 | 6.715 |
| Foxc1 | 6.915 | Cask | 6.693 |
| Hsf2 | 6.899 | Raf1 | 6.662 |
| Tcof1 | 6.846 | Trp73 | 6.66 |
| Slc39a13 | 6.839 | Apaf1 | 6.658 |
| Col2a1 | 6.829 | E2f5 | 6.579 |
