# Supplementary material for: Whole exome sequencing identifies MRVI1 as a susceptibility gene for moyamoya syndrome in neurofibromatosis type 1
Source: PLoS One. 2018 Jul 12;13(7):e0200446. doi: 10.1371/journal.pone.0200446 (PMC6042724; doi:10.1371/journal.pone.0200446)
Supplement: S4 Table — (DOCX) [file pone.0200446.s006.docx]

**S4 Table. List of PCR primers with Hg19 genomic coordinates of amplicons for MRVI1 (RefSeq: NM_001098579.2), GUCY1A3 (RefSeq: NM_000856.5), PRKG1 (RefSeq: NM_001098512.2) and ITPR1 (RefSeq: NM_001099952.2).**

| **Name** | **Primer sequence** | **Hg19 coodinates of amplicon** | **Name** | **Primer sequence** | **Hg19 coodinates of amplicon** |
| --- | --- | --- | --- | --- | --- |
| MRVI1_ex01F | CTCTCCTAGCCTGGTTTGACCTCC | chr11:10673477-10673868 | ITPR1_ex11F | CCCTCCTGAACTATGACTTGTG | chr3:4693716-4694000 |
| MRVI1_ex01R | AAACCCTCTGGCCAGCCTAAAA |  | ITPR1_ex11R | TCCCTAAGCCTTTTACAACACG |  |
| MRVI1_ex02F | GAATAAATGCTCTGGCTCCTGTCC | chr11:10655303-10655696 | ITPR1_ex12F | TGTAGAAAGTGAAGGCTTTGGC | chr3:4699749-4700037 |
| MRVI1_ex02R | AGGACCACATCCAGGAGAGGAAAA |  | ITPR1_ex12R | TGGATTCCAAGAAACCTGGTC |  |
| MRVI1_ex03F | TCTTTTCACCTATTTTCAAGTTGTGGA | chr11:10653482-10653672 | ITPR1_ex13F | GACTAGGGGCTATAGAGTAAACCTC | chr3:4702607-4702834 |
| MRVI1_ex03R | CACGCTGCCAGACCTGTCTTT |  | ITPR1_ex13R | CAAAGGAGCCCTTTCATTAGG |  |
| MRVI1_ex04F | GCATGGTATGGCATGACCTCAG | chr11:10651026-10651332 | ITPR1_ex14F | TTAAAGTCTTATCCTTCCCATCC | chr3:4703707-4703984 |
| MRVI1_ex04R | CCTTCCCTCCCGACCCTAGA |  | ITPR1_ex14R | GATGTCAGAAACTCTCAGTGCG |  |
| MRVI1_ex06_07F | AGGGAGATGTGGCTGAAAGCAC | chr11:10649198-10649647 | ITPR1_ex15F | TTCCAGCCTGCTGAACACTG | chr3:4704693-4704957 |
| MRVI1_ex06_07R | TAGGCTTCTAGGAGACTGTGCCCC |  | ITPR1_ex15R | TGAGCTACCACTCATGCTTTATG |  |
| MRVI1_ex08F | CTCACCTGTTTCCCCTCAACCTG | chr11:10647447-10648189 | ITPR1_ex16F | TTCTAATGTAATCCAGCCACCC | chr3:4706717-4707045 |
| MRVI1_ex08R | CTGAGGTCTTCAGCCCAGGGAGT |  | ITPR1_ex16R | GAGGAAAGTTCACAAACTTCAGG |  |
| MRVI1_ex09F | AAGAGAACAGCCCAGCCCAGT | chr11:10645253-10645465 | ITPR1_ex17F | CTCATCAGGAAACATTGCTGC | chr3:4708968-4709293 |
| MRVI1_ex09R | CATTCACCTTCCTTGATCTGGCAC |  | ITPR1_ex17R | TAAGTCCCTGCATCTTACAGGC |  |
| MRVI1_ex10F | aAGCTCCTGAAGCTAGCTGCCAAC | chr11:10631201-10631475 | ITPR1_ex18F | GTGCACTTAAGGAAGAATTGGG | chr3:4711256-4711515 |
| MRVI1_ex10R | TCTGTGTCCCACCTAGAATCCAA |  | ITPR1_ex18R | GAGCACCAACACTGAATGAAC |  |
| MRVI1_ex11F | GGTCATTCAGAGTCACCTTTGGGT | chr11:10628228-10628392 | ITPR1_ex19F | TGTGTCTTTATGCTGAAAGTAAAGC | chr3:4712331-4712668 |
| MRVI1_ex11R | GTTTTGAATAAGCCCAGTCAAGCA |  | ITPR1_ex19R | TCCTAAGTGCCACTGTTTTGG |  |
| MRVI1_ex12F | CAGCCTTGTCCTCTGCAGTAACTC | chr11:10625875-10626152 | ITPR1_ex20F | ACGACCCTTCATTCATCCTG | chr3:4714754-4715134 |
| MRVI1_ex12R | GAGAAGCATGACACCCTGTATGGC |  | ITPR1_ex20R | ATATGGGGCTATCTACTGCCG |  |
| MRVI1_ex13F | TGGGGACTGCTGATTTAGTCCATAA | chr11:10624597-10624865 | ITPR1_ex21F | GAAGACCTCTCTGGGAATAACTTG | chr3:4715827-4716108 |
| MRVI1_ex13R | TGCTTGGGTGATTGCTCTACTTTA |  | ITPR1_ex21R | TCACATATTTTCTATTTGCCCC |  |
| MRVI1_ex14F | GTGCCAACGGAGCCTTTCCT | chr11:10622384-10622664 | ITPR1_ex22F | AACATCAAATGGAATTGAAGGG | chr3:4716682-4716992 |
| MRVI1_ex14R | TCTGACAGCTCTAATCCTAGCCAAGAG |  | ITPR1_ex22R | AGCATAACAGGAAAGGCTGGAG |  |
| MRVI1_ex15F | CTGCTGGTGCCTAGCCTGAAACTT | chr11:10615624-10615801 | ITPR1_ex23F | TGACATATGCCTCTGAGCATTC | chr3:4718234-4718559 |
| MRVI1_ex15R | GTTTGGGTGACCATGGAAACTTCT |  | ITPR1_ex23R | CAGAACAGAGGGACTGGATCTG |  |
| MRVI1_ex16F | CCAGCCCTTCTGAACTATGGCTATT | chr11:10614967-10615214 | ITPR1_ex24F | TTGGCAGAGTCAAGATGGTATG | chr3:4722173-4722441 |
| MRVI1_ex16R | GCCCTAGGACTGAGTACGAAGGAA |  | ITPR1_ex24R | GCTCTCCCCATTTTCCTTAGAG |  |
| MRVI1_ex17F | CAGCTCTGGCTTCTTCCTTCCATA | chr11:10612993-10613221 | ITPR1_ex25F | AGTTCACCAGCAGCATGTTTG | chr3:4722985-4723178 |
| MRVI1_ex17R | CTTTCCACTGCATTTTGGGGATAA |  | ITPR1_ex25R | CATAATAAAGGGAAGGCCTCTG |  |
| MRVI1_ex18F | CCTAATCAGGAATCGGGCCAAA | chr11:10603309-10603624 | ITPR1_ex26_27F | CGTGAGAGGAGGCATTTGTC | chr3:4725014-4725546 |
| MRVI1_ex18R | GCCACTGGTTACTTCCCTAAAGCC |  | ITPR1_ex26_27R | GATGCTCCCTAGTTTCGAGAAG |  |
| MRVI1_ex19F | CTCAAGTCCCCTCCCCTAGGAACT | chr11:10601901-10602220 | ITPR1_ex28F | TTTCTTTCCTAAAGGTCGATGC | chr3:4725875-4726126 |
| MRVI1_ex19R | CTGCCCTGGACTGGCATTTC |  | ITPR1_ex28R | GCTCAAATGTGTGTACCTTCAAC |  |
| MRVI1_ex20F | GCAGAGAGAACTGTGGGTCTGTGT | chr11:10597819-10598200 | ITPR1_ex29F | AATGCATTATAATCCCCTTTGC | chr3:4726648-4726970 |
| MRVI1_ex20R | TGGGGAAAGGGTGGTAGTCTGAGT |  | ITPR1_ex29R | ATCCAGATGTGAAGAGTTTGGG |  |
| PRKG1_ex01F | GTACTTAGCGCCCATTCACTCG | chr10:52751014-52751592 | ITPR1_ex30F | GTGTTGGGTATAGGAGAAGCTG | chr3:4730109-4730374 |
| PRKG1_ex01R | CAGCACGTGATGGGCAGAAATA |  | ITPR1_ex30R | CAAGCTGTTCCTCCTCCCTC |  |
| PRKG1_ex02F | AGAGCTTGTCAGATGTGCCAGT | chr10:52912879-52913172 | ITPR1_ex31F | TGTGTGTTTCAGTGGACTCTTC | chr3:4732732-4733122 |
| PRKG1_ex02R | GTAATGCATTGCCATCTGTGTGC |  | ITPR1_ex31R | TGAAGAGGGTGCAAGTTCATAC |  |
| PRKG1_ex03F | GAGCCTAACACTCCTCTTCACATT | chr10:53227394-53227661 | ITPR1_ex32F | AACCTCTCTCTTCCTCTGTGAATAG | chr3:4735085-4735482 |
| PRKG1_ex03R | ACAACATCTCAAAGGCCTAGGGA |  | ITPR1_ex32R | ACGACAGCACACACCACATAG |  |
| PRKG1_ex04F | GTGGGCTTAGACAATCAACCCC | chr10:53564057-53564529 | ITPR1_ex33F | GGTCCTTGCTGTGAAGTTGAG | chr3:4738747-4739051 |
| PRKG1_ex04R | AAATTCAGGGTGCTGCATTTCA |  | ITPR1_ex33R | TGCACAAAGATGCTACACACAC |  |
| PRKG1_ex05F | TTTGGCAGATTCCTTGTCATGAAA | chr10:53667147-53667395 | ITPR1_ex34F | TCGCAGATTTCTTAATGTTTGG | chr3:4741431-4741699 |
| PRKG1_ex05R | GTGAAAGAAGCCAGCCAAGCAG |  | ITPR1_ex34R | CTCCCAAATTCACATCAAGCC |  |
| PRKG1_ex06F | TGAAGAGGCTGAGCTGTTTGGT | chr10:53814175-53814379 | ITPR1_ex35F | GGGGTCCAGTGGTTCAAGAC | chr3:4744412-4744711 |
| PRKG1_ex06R | TTACTAACCAACCCCAGGCTCC |  | ITPR1_ex35R | GCTCAATAAATGGCAGCCTTAC |  |
| PRKG1_ex07F | TGGAGGTAAGCTCTTGTGGTTT | chr10:53822105-53822564 | ITPR1_ex36F | AGGGCATTACGTCCATCTGTAG | chr3:4747778-4748101 |
| PRKG1_ex07R | ACAGATTCCAAGTCCAGCTGAA |  | ITPR1_ex36R | CACTGCTGTGTGTGGCAATC |  |
| PRKG1_ex08F | AATCACAATGGACACTGTGCTTT | chr10:53893532-53893717 | ITPR1_ex37F | TTTAGGGCAGAAATCAATGTCC | chr3:4751927-4752215 |
| PRKG1_ex08R | TGTGTGTTGCTGATATGAGTGTGT |  | ITPR1_ex37R | CTTTGTGAGGTCATCAAGGTTC |  |
| PRKG1_ex09F | CACTATATCACTGACTAGGTTGCCA | chr10:53921574-53921815 | ITPR1_ex38F | TGCCCTGAAGTATCTTTAACCTG | chr3:4753302-4753643 |
| PRKG1_ex09R | AGTGTTGGGATGTGTCAAGTCCA |  | ITPR1_ex38R | AGTAATGCCTGAGCTAAGGTGG |  |
| PRKG1_ex10F | AGCAAGATGGAATGTAAGCAGGT | chr10:54011208-54011582 | ITPR1_ex39F | GGCTGGCTTGTATTTCCTTTC | chr3:4758988-4759160 |
| PRKG1_ex10R | TGACATGTCTGCCGCAAAGTAT |  | ITPR1_ex39R | ATCACAGGGCTAACTGGGAAG |  |
| PRKG1_ex11F | TGCACTCTTACAAGTCTATGGGCT | chr10:54031056-54031289 | ITPR1_ex40F | TGTTGGTCTCTGGACTTCTCTG | chr3:4767153-4767323 |
| PRKG1_ex11R | TTGGAGTTGGGTACGTCTGTCC |  | ITPR1_ex40R | ACTGAGAGGCATCCATATTTGC |  |
| PRKG1_ex12F | AAATCAAAACTATAATCTGGGCCCC | chr10:54032060-54032514 | ITPR1_ex41F | TGACTGATGCTGCAGATGGTAG | chr3:4768741-4768917 |
| PRKG1_ex12R | TTCATGCAAAACTAAGGCCATGT |  | ITPR1_ex41R | ATGGAGGTGTTACGGTCATTG |  |
| PRKG1_ex13F | AGCTAGCAGGACAGTGATCTCT | chr10:54040421-54040952 | ITPR1_ex42F | TGTTTTGAATATTCGTCCCTCG | chr3:4774701-4774963 |
| PRKG1_ex13R | TGCCAGCAAGTGTGTTTTCTCA |  | ITPR1_ex42R | GCAGCTATCACAGAACACACTTG |  |
| PRKG1_ex14F | AGGGAGACTGTCTTTGCAATATAGA | chr10:54041749-54042140 | ITPR1_ex43F | ATGCAGCAAACATTAGCTGTTC | chr3:4776788-4777104 |
| PRKG1_ex14R | AAGCAGGAGTGTGTAGCTGCAT |  | ITPR1_ex43R | CTCCCAGAACAGACTCTCCTCC |  |
| PRKG1_ex15_16F | AAGGGCTTCCACACTGTTTTGC | chr10:54048320-54048887 | ITPR1_ex44F | GTTTTGGTGTCATGAGTGGG | chr3:4808141-4808455 |
| PRKG1_ex15_16R | AGCCTTACTAGTTTATACGCTGTCT |  | ITPR1_ex44R | GGATAACAAGGACTCTTCTGGTG |  |
| PRKG1_ex17F | TGTATACACTGCAATGAGAAGCTTT | chr10:54049915-54050112 | ITPR1_ex45F | TTTCTGAGTGTCACCTTTGGAG | chr3:4810115-4810524 |
| PRKG1_ex17R | TGTTGACTGACATAAACACCATCAA |  | ITPR1_ex45R | GCTGAACCATCAGAGGAAGG |  |
| PRKG1_ex18F | ACTGTGACCCTCAATACCTGCT | chr10:54053418-54053764 | ITPR1_ex46F | ATGGCAGGATGAATAACGTTTC | chr3:4816854-4817183 |
| PRKG1_ex18R | GGTGACCCCGAGCACTAATCTT |  | ITPR1_ex46R | CAACATTTCCAAAATGGGAC |  |
| GUCY1A3_ex01F | GTGTCCTTGAATTGATAGTGGC | chr4:156617923-156618359 | ITPR1_ex47F | GGCCTTTGACGTCTGATTTATG | chr3:4818886-4819123 |
| GUCY1A3_ex01R | GGAAGTGTTTAAATTTTCCTTGG |  | ITPR1_ex47R | GCCAAGGCAAAACACAAAAG |  |
| GUCY1A3_ex02F | GAAAATCACTTCCAGTTTCCAAAGT | chr4:156624914-156625213 | ITPR1_ex48F | TGAAATGTTCGTCTGTTTAGCC | chr3:4821119-4821398 |
| GUCY1A3_ex02R | GCTGGCCATTAGTAGCAGTATT |  | ITPR1_ex48R | CCTCTGCTCTGTCCCAAATATC |  |
| GUCY1A3_ex03F | TCCTGACCTTGTGATCCACC | chr4:156629215-156629509 | ITPR1_ex49F | CAGTCCTGTGTGGGTCTTCC | chr3:4824245-4824485 |
| GUCY1A3_ex03R | GGAGAAAATGTGAGTTCTACAGG |  | ITPR1_ex49R | GCTCGGAATTTTGGACCTGTAG |  |
| GUCY1A3_ex04aF | ATGGCACAGCTTTACCAAGG | chr4:156631632-156632101 | ITPR1_ex50F | TTCTGTGTTCCTGTTGTGAAGAG | chr3:4825441-4825661 |
| GUCY1A3_ex04aR | TGCTTTTCATGTGAACGGAG |  | ITPR1_ex50R | CATTGCACACTAATGGCCTC |  |
| GUCY1A3_ex04bF | TGAAACGGAAGTGGAAGTGTC | chr4:156631998-156632476 | ITPR1_ex51F | ACTCAATCTTGACCACCGAGTC | chr3:4829534-4829889 |
| GUCY1A3_ex04bR | TTTTACCCTTTTGTTTCTTGCTC |  | ITPR1_ex51R | AAGCCCAAGTTTGTGAAATCC |  |
| GUCY1A3_ex05F | CTTCCCCTTCTTTTGTCTCAG | chr4:156634177-156634796 | ITPR1_ex52F | AAAGTGGACTTGTGGGGCAG | chr3:4836646-4836939 |
| GUCY1A3_ex05R | ACAGTGCTTGTTCCCCAGTG |  | ITPR1_ex52R | AACTGAGATGCAAGTCCTAGCC |  |
| GUCY1A3_ex06F | GGCTGTGATTCTTCCTGAGC | chr4:156638256-156638528 | ITPR1_ex53F | CATGTTTTAGGTCTGTCCCCTG | chr3:4842037-4842384 |
| GUCY1A3_ex06R | CACCATGGTTTTGGTAATCAAC |  | ITPR1_ex53R | TCTGGACTGAAAGCACAGGC |  |
| GUCY1A3_ex07F | TGACGAGTAGGAAGTGTTTTACC | chr4:156643131-156643423 | ITPR1_ex54F | TGCTCTCATGAAGAGTTTGGC | chr3:4847726-4848030 |
| GUCY1A3_ex07R | ATGAATCAAATCAGTGGGGC |  | ITPR1_ex54R | GGGTCTGTGATGAGAGAGAGG |  |
| GUCY1A3_ex08F | TGGGAAGATGTCTGTTAATCTCG | chr4:156651026-156651460 | ITPR1_ex55F | TAAACCAAGTTTGCATTATGGG | chr3:4852869-4853243 |
| GUCY1A3_ex08R | CCCTAAAGTGCTTTCAGAGGC |  | ITPR1_ex55R | GGAGATATCTGCATTACTAAAGTCG |  |
| ITPR1_ex03F | CCCCTTCTGAACATTTCTTTTC | chr3:4558105-4558371 | ITPR1_ex56F | GGGGACTTCAAACATTTTAACC | chr3:4854758-4854979 |
| ITPR1_ex03R | CCAAGTAAAACGGTGACGTTC |  | ITPR1_ex56R | ATCAGCTAAGCCTCTGGAGGAG |  |
| ITPR1_ex04F | TTAGATCCATGCATAGGAAGCC | chr3:4562545-4562974 | ITPR1_ex57F | AGATGGCATTCAGGAAACAGG | chr3:4856019-4856331 |
| ITPR1_ex04R | AAAGCCAACCCAATCTTGATAC |  | ITPR1_ex57R | TTTACACTCAACACCGCTGC |  |
| ITPR1_ex05F | CTTTAAGCCTTGGTTTCCTCG | chr3:4669249-4669634 | ITPR1_ex58F | GTCTCACTTGAGCTGTGCCC | chr3:4856654-4856981 |
| ITPR1_ex05R | ACCATTACACCCAGATACCACC |  | ITPR1_ex58R | ATCACACCCTCGCAGTATCC |  |
| ITPR1_ex06F | GCAGTGGGATAGAACACATGG | chr3:4681009-4681212 | ITPR1_ex59F | ACAGGAGTGAAACCACAGCC | chr3:4859659-4859985 |
| ITPR1_ex06R | CTCAGCTGCATTCTTTGTAACG |  | ITPR1_ex59R | TTGCTCAGAGCTGAGTAGGC |  |
| ITPR1_ex07F | GGTGGGAGGAATGTTTGCTAC | chr3:4683675-4684035 | ITPR1_ex60F | TGAGAGTTAGGAAACATGGCAC | chr3:4878353-4878694 |
| ITPR1_ex07R | CTCTGGTTCCAACTTTCAACG |  | ITPR1_ex60R | AGATTTGGTGTGGTGATAGTGG |  |
| ITPR1_ex08F | GGTCAATCCGCAGTCCTTATC | chr3:4685761-4686010 | ITPR1_ex61F | AGTATGCGATTTGACGTACAGG | chr3:4887761-4887980 |
| ITPR1_ex08R | TGAAGTCACTCTCAGCACACG |  | ITPR1_ex61R | CAGAACTCATTAGCCATACCCAC |  |
| ITPR1_ex09_10F | TGGATGACACAGTTGTTGTGAG | chr3:4687013-4687613 |  |  |  |
| ITPR1_ex09_10R | AGGTGGGTATAAGTACTTGTTTGTG |  |  |  |  |
